# Day-night variations in sleep and locomotor activity in lambs during the first 72 hours after birth and following the onset of rumination

**DOI:** 10.1371/journal.pone.0348614

**Published:** 2026-05-08

**Authors:** Younes Beniaich, Hicham Farsi, Manal Larbaoui, Hiba Ouardirhi, Nouha Sbai, Mohammed Piro, Mohamed Rachid Achaâban, Paul Pévet, Etienne Challet, Amal Satté, Khalid El Allali

**Affiliations:** 1 Comparative Anatomy Unit, Department of Biological and Pharmaceutical Veterinary Sciences, Hassan II Agronomy and Veterinary Medicine Institute, Rabat, Morocco; 2 Medicine and Surgical Unit of Domestic Animals, Department of Medicine, Surgery and Reproduction, Hassan II Agronomy and Veterinary Medicine Institute, Rabat, Morocco; 3 Institute of Cellular and Integrative Neurosciences, CNRS and University of Strasbourg, Strasbourg, France; 4 Department of Neurophysiology, Military Hospital Mohammed V, Rabat, Morocco; Belgrade University Faculty of Medicine, SERBIA

## Abstract

The sleep-wake and rest-activity profiles of many species undergoes changes during the development. In ruminants, the onset of rumination-related digestive function is contingent on the attainment of full forestomach maturity. The study aimed to characterise sleep–wake at birth and to assess the rumination onset effects on vigilance-states and rest–activity patterns in lambs. Eight newborn lambs were subjected to two non-invasive polysomnographic (PSG) and behavioural recordings. The first recording was conducted immediately after birth for a duration of 72 h, while the second was performed after rumination onset (1–1.5 months) for an additional 72 h. At birth, lambs exhibited twilight activity, with a peak occurrence during the day-night transitions, resulting in an average of 5.3 ± 0.3 h of daytime activity. Following the onset of rumination, a rise in daytime activity was observed, reaching an average of 7.8 ± 0.3 h. PSG results revealed polyphasic sleep, with lambs spending 58.6% (6.4 h) of the night and 39.6% (4.9 h) of the day asleep. The prevalence of NREM-sleep was observed to exceed that of drowsiness and REM-sleep, both during diurnal and nocturnal periods. Four to six weeks later, rumination occupied 28.6 ± 4.5% of the night and 13.3 ± 2.4% of the day, coinciding with reduced sleep time (from 11.3 h to 6.9 h) due to fewer episodes, without changes in episode duration. All sleep stages were found to be affected, whereas wakefulness remained unchanged. These findings suggest that in neonatal ruminants, rumination may compete with sleep without altering wakefulness, providing new insight into behavioural and vigilance states from suckling to the onset of rumination.

## Introduction

Sleep has been identified in a wide range of animal species [1]. In mammals and birds, two distinct standard stages of sleep have been identified: non-rapid eye movement sleep (NREM sleep) and rapid eye movement sleep (REM sleep). NREM sleep is distinguished by a decline in neuronal activity, the EEG signal, exhibits low activity and high amplitude, minimal or no eye movements (EOG) and low muscle activity (EMG). In contrast, during REM sleep, brain activity reaches a level similar to that observed in waking state, while EOG shows the presence of a characterized rapid eye movements and EMG exhibits a clear muscle atonia [2]. The ubiquity of sleep in the animal kingdom throughout their lifetimes highlights its essential function. Sleep plays a pivotal role in the development of the anatomical and physiological functions of species [3]. The structure of sleep is species-specific and undergoes significant changes throughout the life cycle [1]. Sleep duration also exhibits changes between childhood and adulthood in various species, including humans [4]. It is well established that the postnatal period is characterised by long durations of sleep, particularly REM sleep. This phenomenon is attributed to the rapid brain development that occurs during this period [5]. Studies in rats have shown that deprivation of REM sleep during early development delays maturation of the visual cortex [6] and impairs maturation of the motor system [7]. Additionally, REM provides the neural stimulation needed to develop and prepare the neural circuitry for later higher cognitive processing [8]. An emerging physiological function could potentially have an impact on the architecture of the sleep-wake cycle. Ruminants possess an essential element of digestive function, rumination, which is a distinctive feature exclusive to these species [9]. Following birth, ruminants fed with milk are functionally monogastric and display an underdeveloped forestomach system comprising the rumen, reticulum and omasum. During the initial postnatal period, the abomasum and intestines predominate in terms of digestive function [10]. The rumination function becomes apparent when the forestomach has reached full developmental maturity, with the development of the reticulo-rumen and associated microbiomes [11]. The age of first rumination is species and individual specific, with the key factor in the onset of rumination being the consumption of dry feed [12]. Rumination, which occurs mainly during the nocturnal hours in diurnal ruminants, is associated with the resting position [13–15]. The elevated level of activity concomitant with rumination during the resting period renders it a subject of interest for the study of sleep and the vigilance states. Electrophysiological studies of sleep in ruminants have been used since the 1960s to record the animal’s vigilance states during rest. The majority of sleep studies have focused on adult ruminants, particularly in farmed ruminants, including sheep, goats and cows [16,17].

Sheep have been identified as a reliable model for studying brain function [18,19]. Recording over a 24-hour period has confirmed that sheep are diurnal animals. The total sleep time of sheep is approximately five hours, with a minimal amount of REM sleep of between 1 and 2.5% (≈0.5 h) [17–20]. Sheep primarily engage in ruminating behaviour during their rest periods, with an average duration of 9.6 h per 24-h [21]. Furthermore, during fetal life of the lamb, the proportion of NREM sleep increases from 35% on days <120–44% on the last days just before birth [22]. Conversely, the proportion of REM sleep decreases from 53% to 38% during the same period [22]. After birth, the proportion of REM sleep continues to fall, from 17% at 2 days of age to 7% at 15 days [23]. In contrast to the increase observed during fetal life, NREM sleep after birth decreases from 36% at 2 days to 28% at 15 days [23]. It is important to note that the recording period after parturition in this study was only from 09:00–17:00, and that only two newborn lambs were recorded. In addition, the recording method was invasive [23]. This previous study is the first and sole investigation to look at sleep in ruminants during foetal life and immediately after birth. To the best of our knowledge, the changes in architecture of vigilance states from the perinatal period (i.e., monogastric physiological state) to the period of the onset of rumination (i.e., polygastric state) have not been investigated yet. Using a non-invasive PSG (EEG, EOG and EMG) in combination with an ethogram, the present study sought to address this gap in knowledge of ruminants’ sleep. The objective was to characterise the architectures of the sleep and states of vigilance as well as the activity and rest behaviours exhibited by newborn lambs following birth and after the onset of rumination.

## Materials and methods

### Experimental animal model and housing environment

The present study was conducted in the laboratory of the Comparative Anatomy Unit, Hassan II Agronomic and Veterinary Institute, Rabat, Morocco (latitude: 34°01′ N, 6°50′ W). The study was conducted on eight Sardi lambs (three males and five females), a local Moroccan breed, that were born at spring. The pregnant ewes were accommodated in a large over-topped enclosure (100 m2) that incorporated shaded areas, where they were permitted to move freely under natural environmental conditions (photoperiod and temperature), and were fed a concentrated feed (Alf Sahel, Morocco) twice a day (09:00 and 18:00), with straw and water provided by free access. After parturition, the lambs were born healthy and received the necessary amount of colostrum from their mothers. The lambs were not separated from their mothers, with whom they remained in contact at all times. The newborn lambs were examined and found to be in good health, with weights ranging from 3.3 to 3.9 kg at the onset of the experiment, and from 8.3 to 11.0 kg y at the end of the experiment. During the behavioural and PSG recordings periods, each lamb and its mother were separated from the group and placed in a small 15m² enclosure.

All procedures were carried out in accordance with the ethical standards of the European Union Directive 2010/63/EU. This approach is in accordance with current guidelines for ethic in chronobiology studies [24]. All experimental protocols were approved by the local ethics committee for animal science and health and veterinary public health (CESASPV) of the Hassan II Agronomic and Veterinary Institute (IAV), Rabat Institute, Morocco (**Ethics approval number: CESASPV_2025_A05**).

### Experimental design

Following the provision of the requisite amount of colostrum in the initial hours post-partum, each lamb was promptly subjected to two periods of behavioural and polysomnographic recording. The first, designated as “after birth”, was conducted immediately during the perinatal period, while the second period, termed “after onset of rumination”, was initiated after weaning and when lambs have acquired the rumination function. In each of the two periods, behaviours and polysomnographic recordings were performed for 72 hours starting at 22:00.

### Behavioural recording

In each period, lambs’ behaviours were recorded using high-resolution Dahua® HACHFW1200RM cameras. These cameras were equipped with infrared emitters and manufactured by Dahua Technology Co., Ltd in Zhejiang, China. The videos were then stored in a digital video recorder (Dahua DVR (DHIHCVR5108H-S2, Dahua Technology Co., Ltd, Zhejiang, China) with a capacity of 4 terabytes. The cameras were positioned at a height of 3 metres in order to ensure optimal visibility and comprehensive coverage of the enclosure area. This configuration made it possible to track the lamb’s movements with accuracy.

The behaviours of each lamb were scored using the validated video-locomotion scoring technique developed for adult goats and dromedaries [25–27], which was adapted for use with newborn lambs. In the present study, as the primary focus was on sleep, it was deemed necessary to incorporate additional scores for resting positions. A score was assigned to each behavioural level over a thirty-second interval, based on a seven-level rating scale with variable amplitudes (see Table 1).

**Table 1:** Ethogram of lambs with different postures and level of activity and their corresponding scores.

Postures	Scores
Standing mobile (in motion)	6
Standing motionless, moving head	5
Standing motionless, head motionless	4
Sternal decubitus, head up, mobile	3
Sternal decubitus, head up, motionless	2
Sternal decubitus, head down on floor or on flank	1
Lateral decubitus	0

### Polysomnographic recordings

**Fig 1:**
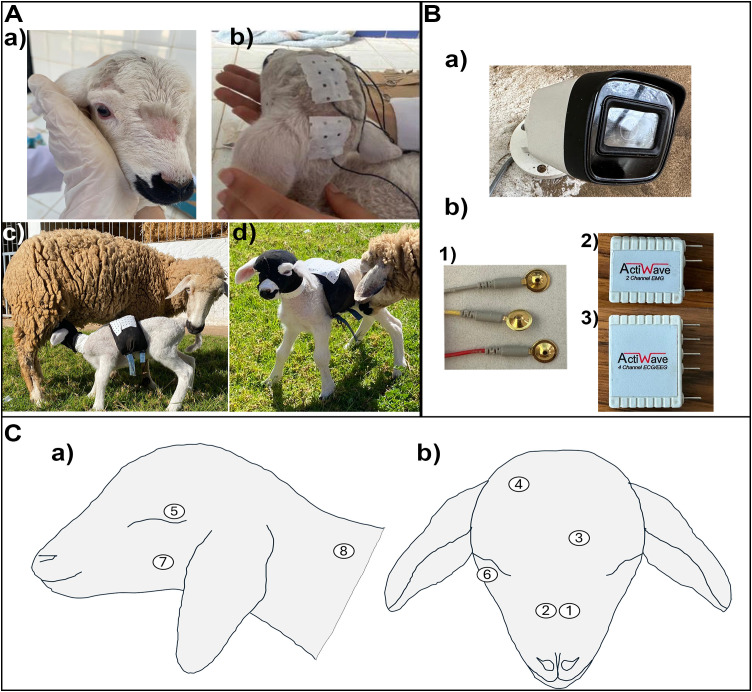
Behavioural and polysomnographic recording of sleep in newborn lambs. Newborn lambs were used in the study (A), the electrode sites were shaved (A, a) and after the electrodes were applied using a medical plaster (A, b), all equipment was protected by handmade protective fabric (A, c and d). High-resolution camera equipped with an infrared emitter to monitor the lamb’s behaviour (B, a). Gold-plated disc electrodes (B, b,1) were used to record EEG, EOG and EMG. These electrodes were connected to two separate devices: the two-channel Actiwave EMG device (B, b, 2) and the four-channel Actiwave EEG/ECG device (B, b, 3) (CamNtech Ltd in Cambridgeshire, UK). **C.** Demonstration of electrode placement on the head and neck of the lamb.

Non-invasive PSG was performed using two miniature ambulatory polysomnography devices: the four-channel Actiwave EEG/ECG device and the two-channel Actiwave EMG device (CamNtech Ltd, Cambridgeshire, UK) (see Fig 1B). Before setting up the recording system, the eight sites on the lambs’ necks and heads were meticulously degreased with alcohol and rubbed with an abrasive gel (Everi®, SPES Medica, Genova, Italy). Gold-plated disc electrodes (CamNtech Ltd, Cambridgeshire, UK) were then attached to the skin using lic2® electrode cream (CNSAC, Germany) and covered with rubber. A medical plaster was subsequently placed over the top to secure the electrodes. Electrodes were positioned on the parietal and frontal cortex of the lambs to record cortical activity (EEG). Ocular activity (EOG) was recorded by two electrodes: one positioned close to the upper eyelid of the right eye and the other near the lower eyelid of the left eye. The EMG electrodes were placed at the level of the masseter muscle and on the splenius musculature of the neck. Two reference electrodes were placed at the level of the nasal bone (Fig 1A and 1C). The equipment is protected by a locally manufactured nylon mask, and the electrode cables are wrapped in plastic sheaths (cannulas) for protection. These pass through the upper part of the animal’s neck and back before being connected to the Actiwave devices, which are placed in protective plastic housings and then inserted into specially designed compartments inside the locally manufactured harness (Fig 1A). The data were sampled at a rate of 256 Hz for EMG, and 128 Hz for EEG and EOG at 10-bit resolution, and stored on the device until the end of each recording session, at which point they were downloaded to a computer.

### Data analysis

#### Behavioural data.

Behavioural recordings were made over a period of three consecutive days during each phase for each lamb. Actograms were constructed to show the distribution of behavioural score data over 24 hours using GraphPad Prism 8.0.2 software.

The quantity of behaviour scores was measured separately for each lamb over a 30-second interval. The inactivity/activity threshold was delineated when lambs transitioned from score 3 to score 4. Scores of 4, 5 and 6 were then utilised to calculate activity. The duration of activity in minutes for each hour was calculated for each lamb and then averaged. Additionally, the means (± SEM) of activity and inactivity times in hours were calculated independently for night, day, and the 24-hour period. Paired t-test was performed to determine significant differences in the amount of activity and rest during and between each period: at birth period and after onset of rumination period. A significance level of p ≤ 0.05 was used to indicate statistical significance.

#### Polysomnographic data.

The duration of the recording period was three consecutive days for each lamb in both periods (at birth and after onset of rumination). The PSG data (EEG, EOG and EMG) were downloaded from the Actiwave devices as EDF files and analysed visually using Polyman®, a freely available sleep assessment program. The signals were manually blind-scored in 30-second periods and displayed on fixed scales of 100 μV for EEG and EMG and 250 μV for EOG. Prior to scoring, both EEG channels were digitally filtered using a band-pass filter with a high-pass cut-off of 0.3 Hz and a low-pass cut-off of 50 Hz. The vigilance states recorded comprised wakefulness, drowsiness (light sleep), NREM sleep (deep sleep), REM sleep and rumination, which began during the second period. Hypnograms illustrating the distribution of vigilance states over each recording period were generated using GraphPad Prism 8.0.2 software.

The mean values (± SEM) were calculated for the **percentage of time spent in each vigilance state**, both at night and during the day. Additionally, the means (± SEM) of the **number and duration of episodes for each vigilance state** at night and during the day were calculated. Furthermore, the ratio **of total sleep time (TST)** – defined as the sum of drowsiness (light sleep), NREM sleep (deep sleep), and REM sleep – was determined separately for night and day. Finally, the **percentage occurrence of each vigilance state in each behavioural score**, was calculated and averaged for each lamb in each period separately. Paired t-tests and one-way analyses of variance (ANOVA) with repeated measures (RM) were performed to determine significant differences in the mean values of the specified variables within or between the two experimental periods, with a significance level of p ≤ 0.05 indicating statistical significance.

## Results

### Daily pattern of rest-activity

**Fig 2:**
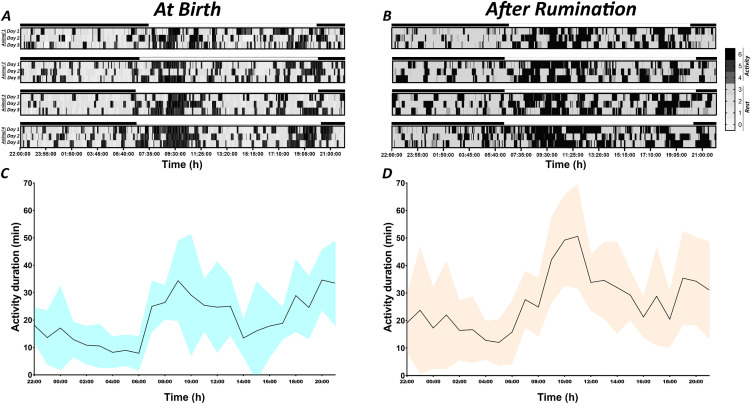
Locomotor activity patterns in lambs at birth and after the onset of rumination. Actograms illustrative of the locomotor activity of four representative lambs, at birth (A) and after rumination (B). The duration of activity (min) was measured every hour for each animal and then averaged for all lambs (C: at birth and D: after rumination). The intensity of the grey colour increases with increasing activity level. The black and white bars situated at the top of the actograms correspond respectively to the dark and light phases of the natural LD cycle.

In the present study, eight lambs were observed over the course of two periods each of three days, during which behavioural and polysomnographic recordings were conducted. The results of a visual inspection of the actograms showed that newborn lambs after birth exhibited a twilight pattern of activity, with a peak of activity during the day-night transition and a second peak before night-day transition (Fig 2A). The two peaks of activity occurred between 06:00 and 14:00 and between 17:00 and 23:00, respectively (Fig 2C). The activity duration at each of these peaks was higher and exceeded 20 minutes per hour (Fig 2C). Conversely, the onset of rumination in lambs resulted in a marked increase in activity during the day, thereby establishing a transition from a bimodal to a unimodal diurnal activity pattern. This shift in activity pattern is characterised by a duration of activity exceeding 20 minutes/hour from 07:00–22:00 and reaching 50 minutes between 10:00 and 11:00 (Fig 2B and 2D).

**Fig 3:**
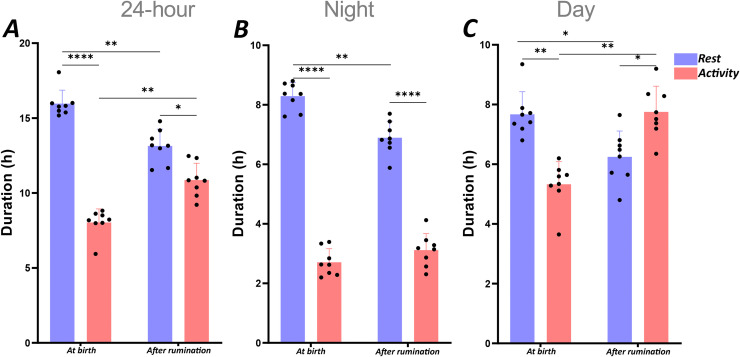
Duration of rest and activity periods. Mean (± SEM) of total duration of rest and activity during the 24-hour (A), Night (B) and Day (C) of each recording period. The duration of rest (scores 0, 1, 2 and 3) and activity (scores 4, 5 and 6) were calculated during the 24-hour, Night and Day periods during each phase. Significance levels were set at p < 0.05 (*), p < 0.01 (**), p < 0.001 (***), p < 0.0001 (****).

A quantitative analysis of rest (scores 0, 1, 2 and 3) and activity (scores 4, 5 and 6) over a 24-hour period was conducted, revealing a significant predominance of rest in both periods, during suckling and after onset of rumination (Fig 3A). A paired t-test analysis revealed that these differences between durations of activity and rest are significant within each period (p < 0.0001: at birth; p = 0.0242: after onset of rumination) (Fig 3A). Furthermore, the onset of rumination significantly (p = 0.0026) reduced the amount of rest from 15.9 ± 0.3 h to 13.1 ± 0.4 h and significantly (p = 0.0025) increased activity from 8.1 ± 0.3 h to 10.9 ± 0.4 h (Fig 3A).

During the night-time period, the dominant state was rest (Fig 3B). A significant decrease (p = 0.0014) in rest was observed when transiting from the period of birth (8.3 ± 0.2 h) to period after onset of rumination (6.9 ± 0.2 h) (Fig 3B). Such differences were not observed for the duration of activity which remained stable in the perinatal period and after acquisition of the rumination function (p = 0.1790).

In contrast to nighttime, the analysis during daytime revealed a complete inversion of the proportion of rest and activity when transiting from the period following birth to the period after onset of rumination. Indeed, after birth, the animals exhibited a greater propensity for daytime rest (7.7 ± 0.3 h) in comparison to daytime activity (5.3 ± 0.3 h) (p = 0.0033); However, when the rumination function has been acquired, lambs displayed a marked decrease in daytime rest (6.2 ± 0.3 h) in comparison to the daytime resting at birth (7.7 ± 0.3 h) (p = 0.0222). Comparison of at birth and rumination periods has shown statistical differences in the duration of activity which increased (p = 0.0016) (Fig 3C).

### PSG vigilance states

The perinatal period was characterised by four vigilance states: wakefulness, light sleep (drowsiness), deep sleep (NREM sleep) and REM sleep. While, in the second period, 4–6 weeks later, there was an emergence of a new distinct state, the rumination, which is characterised by specific and rhythmic spike-like EMG activity and myogenic artefacts on EEG and EOG.

#### Vigilance states.

**Fig 4:**
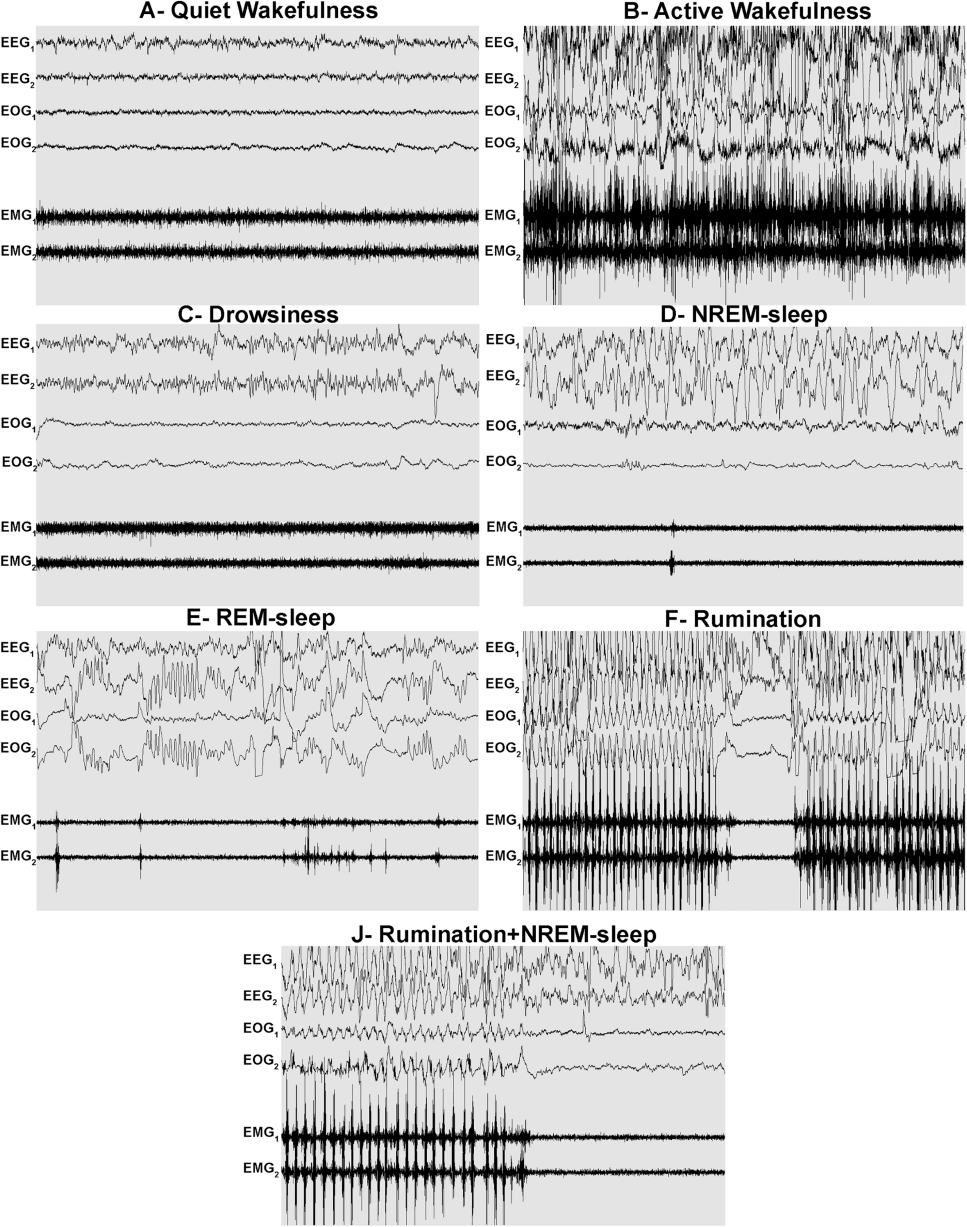
Polysomnographic traces of states of vigilance in newborn lambs. A- quiet wakefulness, B- active wakefulness. C- drowsiness (light sleep). D- NREM sleep (deep sleep). D- REM sleep. E- Rumination. F- Rumination+NREM sleep. EEG waveforms from frontal and parietal electrodes are represented by EEG1 and EEG2, respectively. EOG1 and EOG2 are the electro-oculogram waveforms of the right and left eye electrodes, and EMG1 and EMG2 represent the electromyograms of the splenius and masseter muscles, respectively. PSG signals were recorded in 30-second periods.

During **quiet wakefulness** (see Fig 4A), the electroencephalogram (EEG) exhibited high frequency and low amplitude, corresponding to Beta waves (from 14 to 30 Hz) in the frontal and parietal regions of the brain. The electrooculogram (EOG) revealed the presence of ocular movements, including blinking and voluntary eye movements. The electromyogram (EMG) recorded at the splenius level revealed elevated muscle tone, characterised by high amplitudes and frequencies.

**Active wakefulness**: In the case of active wakefulness (Fig 4B), there is an important increase in muscles activity with significant myogenic artefacts that are related to the animal’s voluntary movements influencing EEG and EOG tracings.

**Drowsiness/Light-Sleep**: the transition from wakefulness to sleep is characterised by a transitional phase known as drowsiness (Fig 4C), during which brain activity is reduced compared to the waking state but remains higher than that observed during deep sleep. The EEG during drowsiness shows mainly low-amplitude waves of higher frequency than in NREM sleep, or Theta waves (frequency 4–7 Hertz). In certain instances of drowsiness, the presence of “K-complex” waves becomes evident on the EEG. EMG muscle tonic activity is lower than in the awake state. The EOG may demonstrate sporadic slow eye movements.

**NREM sleep/Deep sleep**: the NREM sleep stage is characterised by a reduced tonic activity in comparison to the levels observed in drowsiness and wakefulness, with muscle movement artefacts being infrequent or absent (Fig 4D). EEG analysis reveals a low-frequency, high-amplitude signal composed of delta waves (0.5 to 4 Hz), indicative of the slow-wave sleep phase. Eye movements are absent.

**REM sleep**: characterised by the presence of rapid horizontal eye movements. These are defined as a succession of eye movements of high amplitude, exceeding 120 µV, and with a frequency of 3–7 eye movements per second. This enables a clear distinction between this stage and other vigilance states. In comparison to other sleep stages, REM sleep exhibits reduced tonic muscle activity, occasional bursts of EMG muscle activity (Twitch), and brain activity resembling to wakefulness (Fig 4E).

**Rumination**: This vigilance state is absent in the newborn lamb and appears only 4–6 weeks later. It is characterised by fast, rhythmic myogenic depolarizations in the EMG, each lasting for 0.5–0.8 sec, with a high mean amplitude of 325.2 ± 0.2 μV generated by regular muscle twitching of the masseter and correspond to chewing. This process has been observed to induce myogenic artefacts affecting EEG and EOG signals. Consequently, these artefacts manifest as a rapid, rhythmic trace having a specific pattern of spike-like waves (Fig 4F).

**Rumination-NREM sleep**: After onset of rumination, it was possible in certain instances to obtain tracings of the initial rumination episodes in which the EEG tracings exhibited concomitant slow-waves sleep (SWS) of NREM (Fig 4J).

#### Vigilance states architecture.

**Fig 5:**
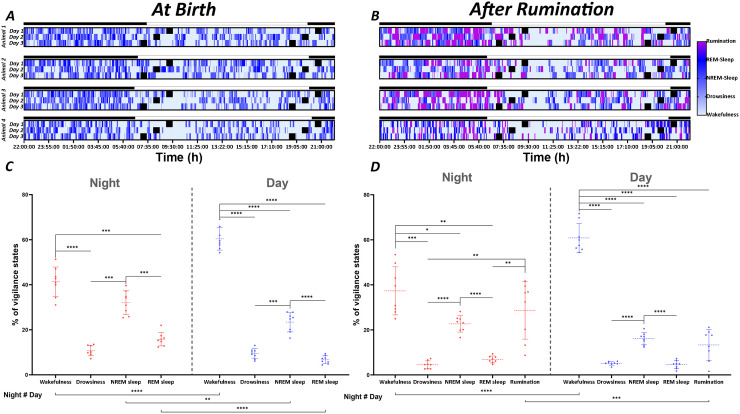
Distribution of vigilance states. Hypnograms representative of the two recording periods (A and B), presenting the distribution of vigilance states (wakefulness, drowsiness, NREM sleep, REM sleep and rumination) during the recording periods illustrated. The increase in colour density indicates the transition from wakefulness to sleep stages, with the black and white bars corresponding to the dark and light phases of the L/D cycle, respectively. The mean (± SEM) proportion of vigilance states was calculated and averaged over the night and day of each recording period (C and D). Significance levels were set at p < 0.05 (*), p < 0.01 (**), p < 0.001 (***), p < 0.0001 (****).

**At birth:** As illustrated in Fig 5, A and B, the hypnograms demonstrate the distribution of different vigilance states throughout the day and night in representative newborn lambs following birth (Fig 5A). At birth, the lambs tend to sleep (drowsiness/light sleep, NREM/deep sleep and REM sleep) with polyphasic episodes both during the day and the night, but with an almost dominance in the nocturnal phase. In contrast, during the day, although that episodes of sleep are important, they are interrupted with extended periods of wakefulness (Fig 5A). This finding was corroborated by a quantitative analysis of the proportions of vigilance states in Fig 5C. Indeed, lambs at birth spent 58.6% of the night-time asleep, while they spent 41.4% of the nighttime awake (Fig 5C). During the day, the proportion of time spent awake exceeds 60%, while the proportion of time spent asleep is less than 40% (Fig 5C). The RM one-way ANOVA for the different vigilance states during the nocturnal recording period demonstrates a significantly higher proportion of wakefulness (41.4 ± 2.3%) and NREM/deep sleep (32.1 ± 1.9%) in comparison to drowsiness/light sleep (p < 0.0001; p = 0.0002, respectively) and REM sleep (p = 0.0002; p = 0.0002, respectively), which respectively present 10.7 ± 0.8% and 15.8 ± 1.1% (Fig 5C). As for night-time, wakefulness during daytime, occupied the highest proportion (60.4 ± 1.8%) compared with the percentages of drowsiness, NREM sleep and REM sleep (9.4 ± 0.8%; 23.4 ± 1.5% and 6.7 ± 0.6%, respectively) and these differences were found to be highly significant (p < 0.0001; p < 0.0001; p < 0.0001) (Fig 5C). NREM sleep during daytime was also significantly higher compared with drowsiness and REM sleep (p = 0.0005; p < 0.0001). During the night, total sleep time (TST) represents a majority proportion (58.6%) of time, significantly dominating wakefulness (41.4%). In contrast, an opposite trend is observed during the day, when wakefulness predominates (60.4%) over sleep time (39.5%).

**After the onset of rumination:** Visual observation of the hypnograms revealed that the onset of rumination in lambs affected the distribution of wakefulness and sleep states. The prevalence of rumination was more pronounced during night than during day (Fig 5B). It is noteworthy that rumination exerts an influence on sleep states, with a minimal effect on the wakefulness state. As demonstrated in Fig 5D, during the night, wakefulness and rumination collectively account for the majority of the recording time, with 37.3 ± 3.8% and 28.6 ± 4.5%, respectively. Conversely, drowsiness (light sleep), NREM (deep sleep) and REM sleep states collectively account for 4.5 ± 0.6%, 22.7 ± 1.3% and 6.9 ± 0.5%, respectively. RM one-way ANOVA analysis revealed that during night, the proportion of wakefulness was found to be statistically higher than that of other sleep states (drowsiness, NREM sleep, REM sleep) (p = 0.0003; p = 0.0195; p = 0.0011). The proportions of rumination and NREM sleep were significantly higher than those of drowsiness and REM sleep (p = 0.0098, p = 0.0098; p < 0.0001, p < 0.0001) (Fig 5D).

During the day, the proportion of wakefulness (60.8 ± 2.3%) was found to be statistically higher than the proportions of drowsiness (5.1 ± 0.3%), NREM sleep (16.1 ± 0.9%), REM sleep (4.6 ± 0.6%) and rumination (13.3 ± 2.4%) (p < 0.0001, p < 0.0001, p < 0.0001, p < 0.0001). In addition, NREM sleep was significantly higher than drowsiness and REM sleep (p < 0.0001, and p < 0.0001) (Fig 5D).

#### Comparison of PSG states at birth with period after onset of rumination.

**Fig 6:**
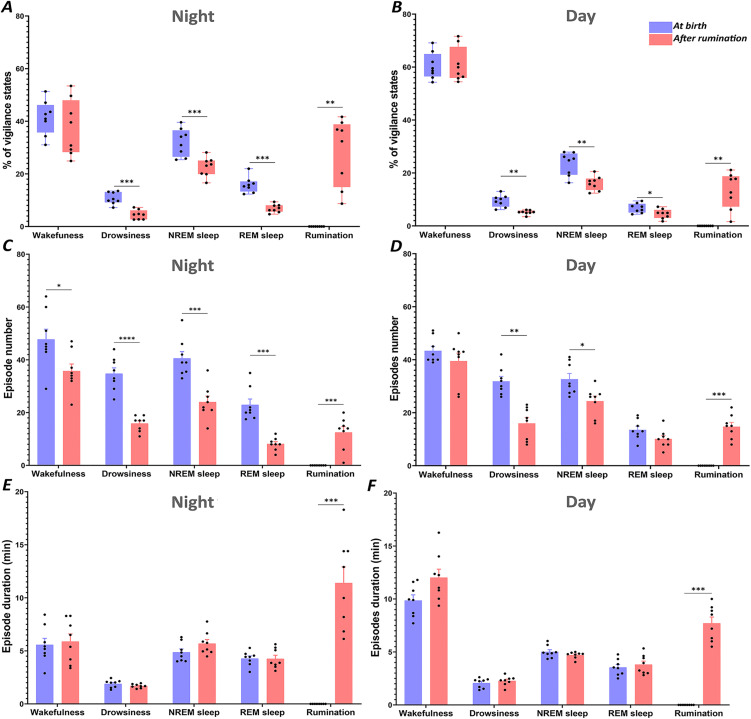
Vigilance state architecture. Comparison of the mean (± SEM) percentage of time spent in each vigilance state during night and day (A and B), the mean (±SEM) number of episodes of each vigilance state (C and D) and the mean (± SEM) duration of episodes of each vigilance state (E and F), recorded during the first recording period (at birth) and the second recording period (after rumination). The percentages of each vigilance state, duration and number of episodes were calculated for each animal and then averaged for all animals separately for night and day. The significance levels were set at p < 0.05 (*), p < 0.01 (**), p < 0.001 (***), and p < 0.0001 (****).

**Vigilance states duration:** The rumination function was found to occur at the expense of sleep stages during both the night and the day (Fig 6). At night, the effect is prominent (Fig 6A). Indeed, paired t-test analysis revealed a significant decrease in percentage of drowsiness (light sleep), NREM sleep (deep sleep) and REM sleep from 10.7 ± 0.8%, 32.1 ± 1.9% and 15.8 ± 1.1%, respectively, at birth to 4.5 ± 0.6%, 22.7 ± 1.3% and 6.9 ± 0.5%, respectively, after the onset of rumination (p = 0.0003; p = 0.0009; p = 0.0001, respectively). Wakefulness proportions remained unaltered by the onset of rumination.

**Episode number and duration of vigilance states:** A subsequent quantitative analysis in the lambs showed that the onset of rumination 4–6 weeks after birth affects significatively episodes number of the other vigilances states but not their episode duration (Fig 6). Indeed, during the night-time a decrease in the number of episodes of drowsiness (light sleep), NREM sleep (deep sleep) and REM sleep has been observed, from 34.7 ± 2.2, 40.6 ± 2.5, and 22.9 ± 2.2 at birth to 15.9 ± 1.1, 24 ± 2.2, and 8.1 ± 0.8 after onset of rumination. This decline was found to be statistically significant (p < 0.0001, p = 0.0002, p = 0.0003) (Fig 6C). During the daytime recording period, a similar significant decrease was only observed for the number of drowsiness (light sleep) and NREM sleep (deep sleep) episodes, from 31.8 ± 1.8 and 32.7 ± 2.1, respectively, at birth to 16 ± 2.2 and 24.4 ± 1.9, respectively after rumination (p = 0.0033; p = 0.0432, respectively) (Fig 6D).

The number of rumination episodes showed individual variation, ranging from 1 to 20 episodes, with an average of 12.5 episodes during the nocturnal recording period (Fig 6C).

**Fig 7:**
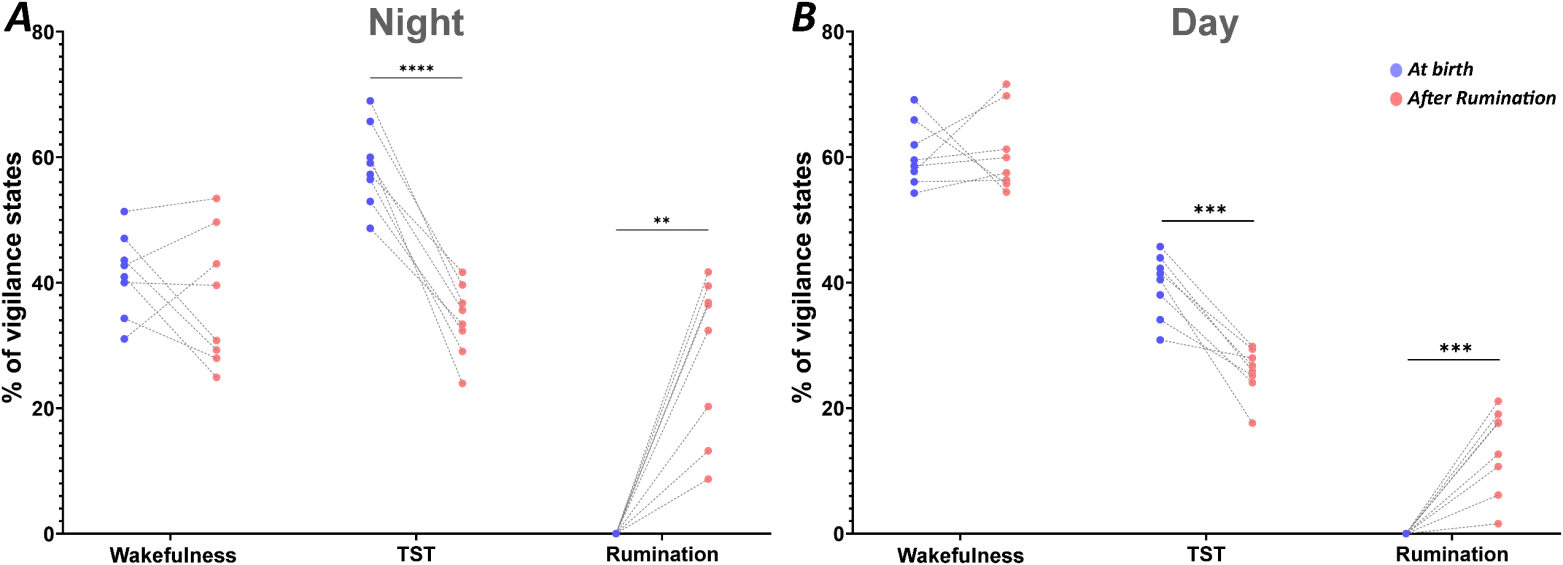
Total sleep duration at birth and after rumination. Comparison of the average percentage (± SEM) of total sleep time (TST) (the combination of drowsiness, NREM and REM sleep percentages) during the night and day of the two study recording periods (at birth vs after rumination). The TST percentage was calculated and averaged separately for the night and day periods. Significance levels were set to p < 0.05 (*), p < 0.01 (**), p < 0.001 (***), p < 0.0001 (****).

**Total sleep time:** A considerable variability in the percentages of wakefulness, total sleep time and rumination states has been observed when comparing the period of birth with the period after the onset rumination (Fig 7). Paired t-test analysis shows that the increase in rumination percentage was accompanied by a significant decrease in TST percentages, from 58.6 ± 0.7% to 34.1 ± 0.3% during the night, and from 39.6 ± 0.2% to 25.8 ± 0.1% during the day, respectively (p < 0.0001; p = 0.0003, respectively). In contrast, wakefulness remained unchanged between the two periods of recordings.

**Fig 8:**
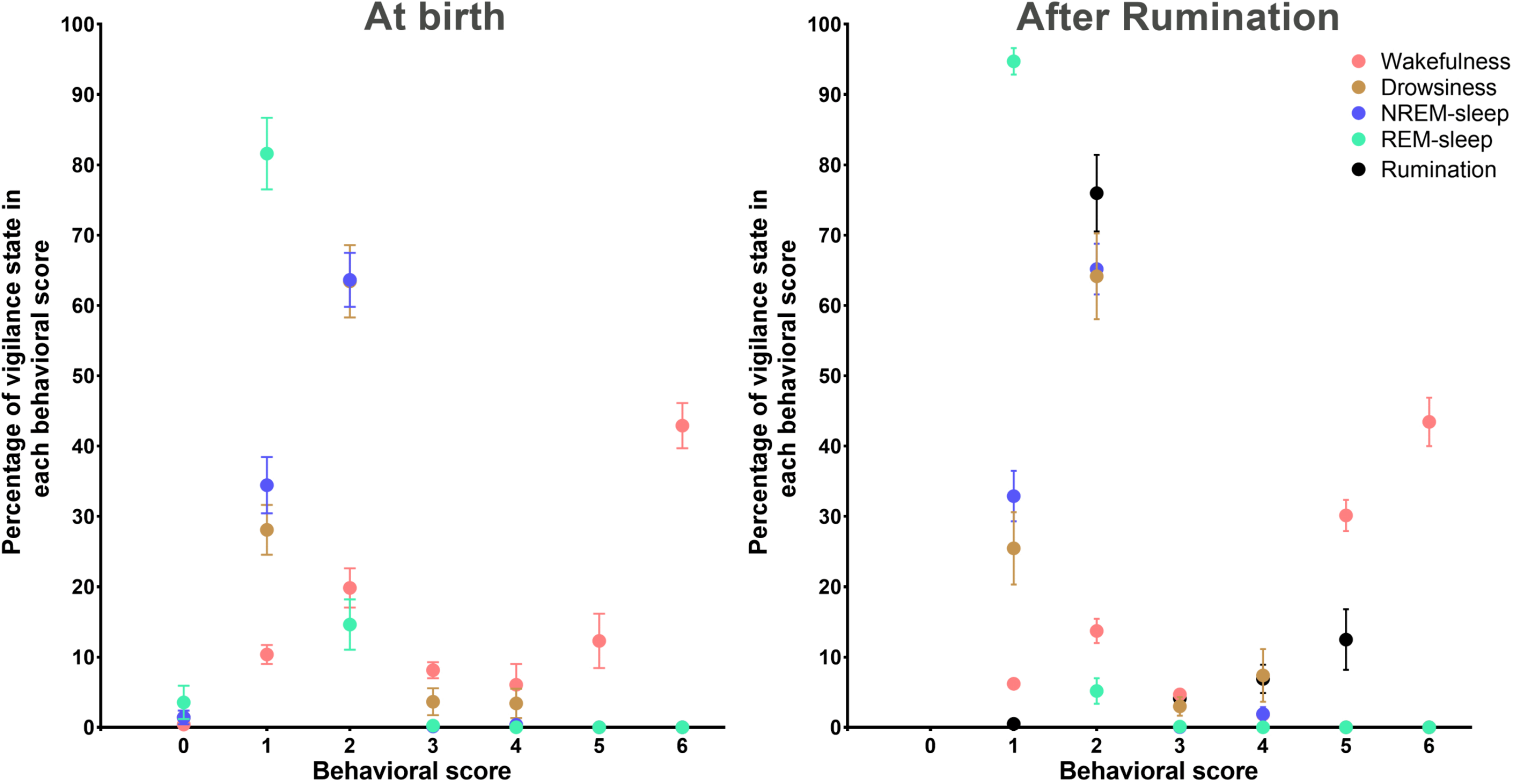
Correlation between behavioural postures and vigilance states. Average percentage (± SEM) of vigilance states in each behavioural score, during the initial period (at birth) and the second period (after rumination).

**Correlation between vigilance states and behavioural position:** As illustrated in Fig 8, a quantitative analysis was conducted to determine the proportion of occurrence of each vigilance state in each behavioural score. At birth, it was evident that the scores allocated to activity (4, 5, and 6) correspond to the awake state, with an exclusivity of this state in scores 5 and 6. The awake state was found to be higher in score 6 (Standing with motion) with an occurrence of 42.9 ± 3.2%, and lower in score 0 (lateral decubitus) having an occurrence of 0.4 ± 0.2%. The majority of REM sleep states (81.6 ± 5.1%), characterised by muscular atonia, were correlated with score 1, which corresponds to sternal decubitus accompanied by head and neck lying on the floor or on the flank. In addition, 3.5 ± 2.4% of REM sleep was found in lateral decubitus. In contrast, the prevalence of drowsiness (light sleep) and NREM sleep (deep sleep) was observed to be significantly higher in behavioural scores 1 and 2, with a predominance found in score 2 (63.4 ± 5.1% and 63.6 ± 3.8%, respectively). The development of rumination in lambs was found to be associated with a specific behavioural state, characterised by rest in sternal decubitus with an erect, immobile head (score 2). It was observed that 75.9% of rumination occurred within this behavioural score. Furthermore, the onset of rumination in newborn lambs did not appear to influence the correlation between the other vigilance states and behavioural postures. Indeed, as for the period after onset of rumination, drowsiness (light sleep) and NREM sleep (deep sleep) in the neonatal phase were similarly associated with behavioural score of 1 and 2, with a predominance found in these scores 64.1 ± 6.1% and 65.2 ± 3.6%. Conversely, lateral decubitus disappeared with the onset of rumination. Furthermore, REM sleep was almost exclusively associated with score 1 (94.7 ± 1.9%). No significant effects were observed for the state of wakefulness, which persisted predominantly in scores 5 and 6, with 43.4 ± 3.4% of wakefulness associated with score 6.

## Discussion

The present study provides a detailed characterisation of activity, behaviour and the architecture of sleep in the newborn lambs during nursing and after the onset of rumination, 4–6 weeks later. A recently born lamb has a bimodal activity with two peaks around sunset and sunrise. The neonate spends much time sleeping and resting. In fact, our study clearly shows that rest was predominant in newborn lambs both during the day and at night. On the other hand, after the onset of rumination observed 1 to 1.5 months later, daytime activity became predominant and lambs began to exhibit a unimodal and diurnal pattern of rest-activity. The study provides also evidence of a polyphasic sleep in the neonate lambs with a surprising predominance of NREM sleep (deep sleep) compared to REM sleep and drowsiness (light sleep). In addition, the onset of rumination led to a significant reduction in total sleep time, but not of wakefulness. This reduction of TST in favour of rumination concerned NREM sleep, REM sleep and drowsiness and is mainly due to a decrease in the number of episodes but not of the episode’s duration. These results are of great interest as they provide new insights into understanding the physiology of sleep in ruminants and the temporal changes in behaviour and vigilance states from birth to the onset of rumination.

### Activity and behaviour in the newborn lambs

Adult ruminants, including sheep, exhibit a diurnal pattern of activity while they tend to rest and sleep briefly during the dark phase [28]. The daily patterns in the lambs of the present study demonstrate that immediately after birth, activity and rest were organized in time over a 24-hour period. Furthermore, actograms highlight that locomotor activity presented a clear pattern with two peaks of activity, one at the sunrise and the second one at sunset. These findings align with the actigraphy observations made in artificially reared lambs and lambs reared by their mothers [29,30]. The duration of rest is higher in almost all mammalian species following birth [5]. In the present study, lambs exhibited a resting tendency for 66% (15.9 h) of the 24-hour after birth, a comparable proportion of rest during the day having previously been observed in weaned dairy calves [31]. In three-day-old piglets, the proportion of the day spent resting (lying down) is almost 70% [32]. In addition, human newborn babies have been observed to spend over 50% of their day in a rested state [33]. Carnivores, such as African lion cubs, have also been shown to dedicate over 60% of their time to rest [34]. The significant amount of rest observed in young herbivores may be attributed to their limited awareness of predation risk at this stage, and to the protection provided by their mothers. Conversely, as herbivores mature, the duration of rest decreases, which may be attributed to the increased vulnerability of ruminants to predation. Consequently, ruminants are compelled to maintain heightened vigilance in exposed environments [35]. In recently born ruminants, the rumen is both physically and metabolically underdeveloped, bearing resemblance to that of a monogastric species [36]. Suckled milk is directedly conducted to the abomasum for digestion. Ruminants undergo significant physiological changes after birth, including rumen development, which involves cell growth and differentiation through the rumen, this dynamic gives rise to the rumination function, which represents a crucial and distinctive aspect of the digestive process in these species [9]. The initiation of rumination in ruminants is contingent on the timing of weaning, with studies showing that early weaning and the provision of starter feed to lambs promote rumen development [37]. In the present study, the lambs were kept with their mothers throughout the experiment, without undergoing weaning or fattening trials. The onset of rumination in the lambs of the present study occurred between 4 and 6 weeks of age, which is consistent with the results of Liu et al. (2022) [37]. It is noteworthy that at this stage, lambs begin to eat food independently. In the present study, lambs tend to become more active as they move around to graze and explore their environment, shifting from an average of 8.1 h of activity when rumination is absent to 10.9 h when rumination appears. This shift towards a diurnal pattern of activity involved a reduction in the amount of rest during the night from 8.3 to 6.9 h, and no effect on the amount of activity during the night. In general, terrestrial mammals undergo a period of maximal rest during the first post-natal days, which subsequently declines as they mature [38]. In the present study, lambs could rest in the lateral recumbency position at birth (1.1% of 24 h), whereas after the onset of rumination, we observed a complete disappearance of this posture (0%). In calves, Pucora and Clauss (2018) observed a decrease in the prevalence of lateral decubitus with age, until sternal decubitus becomes the only lying position in calves at 16 weeks of age [39]. The avoidance of the lateral recumbency in ruminants is related to the gravity gradient of ingesta stratification in the reticulorumen (lighter and larger particles only gather in the dorsal region), both when standing and at rest. Therefore, the relative positions of the in- and outflow orifices of the forestomach remain unchanged. This is due to the digestive anatomy, which generates a constraint that causes ruminants to rest mainly in sternal recumbency and usually does not allow them to enter lateral recumbency [40].

### Polysomnographic aspect of newborn lambs

#### Vigilance states in newborn lambs.

The wakefulness EEG has been reported in earlier studies in neonatal lambs [41], 3-month-old calves [42] and in adult ruminants including sheep [43], cows [44], goats [45], and dromedaries [46,47]. Drowsiness is a prevalent state of vigilance in ruminants, divergent from wakefulness and deep sleep. Following Bell’s observation of this state in goats in 1960 [16], the majority of studies on sleep in ruminants have identified it as a discrete state [17,44,46,48]. However, during this state, specific features have been identified, including an EEG signal pattern showing activity at 3–8 Hz and high amplitude (theta waves), the occasional appearance of the K complex in EEG tracings, or the presence of slow rolling eye movements in the EOG, which are observed in the N1 stage of sleep in humans [49,50]. Consequently, some authors have postulated that the state of drowsiness in ruminants (cow, sheep and dromedary) could be analogous to light sleep (stage N1) [47,51,52]. In the present study, the detection of K-complex and theta waves (4–7 Hz) in the EEG, accompanied by transient slow rolling eye movements, provides arguments to consider this state as a sleep state, termed light sleep.

The characteristics of NREM (deep sleep) and REM sleep in mammals have been extensively documented. In contrast to the higher primates and carnivores, where NREM sleep can be subdivided into multiple stages [53], ruminants do not exhibit such a subdivision of NREM sleep. In ruminants, NREM sleep is distinguished by the presence of SWS, characterised by low muscle activity and the absence of eye movements [17,18,44,46]. The aforementioned characteristics have been observed in newborn lambs in this and other studies [23], as well as in other newborn ruminants such as three-month-old calves [42]. However, in primates, typical NREM sleep patterns such as SWS do not emerge until 2–3 months of age [54]. In the initial weeks of life, the only discernible distinction between sleep stages is between wakefulness, active sleep (REM sleep) and quiet sleep (non-REM sleep) [55]. Quiet sleep (NREM sleep) in neonatal primates is characterised by a low-frequency, high-amplitude signal in the EEG, whereas the classification of NREM sleep is not applicable to human neonates [56]. REM sleep in newborn lambs is characterised by the presence of rapid eye movements, reduced muscle tone relative to other sleep stages, occasional bursts of electromyographic muscle activity (Twitch), and brain activity that resembles that of wakefulness. The EEG signal of rumination exhibits distinct characteristics depending on the methods used to record it. Invasive methods, employing electrodes implanted directly on the surface of the dura mater, have demonstrated that rumination is a physiological phenomenon that can coincide with various other vigilance states, including wakefulness, drowsiness (light sleep) or NREM sleep (deep sleep) [16–18], but ruminants are unable to engage in REM sleep during rumination [17,57,58]. The employment of non-invasive methodologies can complicate the delineation of EEG and EOG patterns, owing to the presence of pronounced myogenic artefacts instigated by mastication during rumination, which impact all PSG channels, culminating in an immutable rhythmic pattern that has been identified in all studies of ruminant species, including dairy cows, deer mice and dromedaries [44,46,48], thereby establishing rumination as a distinct state of vigilance, separate from the other states. The same results concerning the unchanging regularity of rumination were found in newborn lambs using non-invasive methods. However, it should be noted that in the first episodes of rumination, when the chewing mechanism linked to rumination was not strong, changes in the EEG channels of the PSG recording were possible to notice, finding a clear SWS coinciding with myogenic rhythmic artefacts (EMG) corresponding to rumination.

#### Comparison between the period after onset of rumination and at birth.

In adult species, two categories of sleepers have been identified: those who sleep for a single period of the day (classic monophasic sleepers, e.g., humans) and those who sleep for multiple periods during the day (classic polyphasic sleepers, e.g., rats). Ruminant species exhibit a daily rhythm characterised by diurnal activity, with a tendency to sleep for brief periods, concentrated during the dark phase [59]. The organisation of the different states of vigilance in newborn lambs of the present study shows a distribution in the nocturnal and diurnal parts of the day, with a fragmented sleep, accompanied by rapid transitions from one state of vigilance to another, with the condensation of sleep states (drowsiness/light sleep, NREM sleep/deep sleep and REM sleep) during the nocturnal phase. However, these sleep states become less prominent in the second recording phase, when rumination is acquired. Ruminants are generally considered to be prey in to the same way as other herbivores, and sleep has been demonstrated to render these species more vulnerable to predation [35]. This risk has led to the adaptation of these species, whereby they are compelled to maintain heightened vigilance and experience significantly diminishes sleep duration. Adult sheep typically sleep for only five hours per 24-h period [17–20]; while the present results show that in the first three days of age, newborn lambs exhibited a longer sleep duration of 10.3 h. This finding is consistent with the prevailing knowledge among terrestrial mammals, suggesting that sleep duration is highest during the early stages of life [5]. A previous study of sleep on only two lambs revealed that newborns tend to sleep for 7 h in the first 21 h after birth [23].

In contrast to the higher proportion of REM sleep which is well-known in human babies and other newborn species, the present study demonstrates that in the neonate lambs, REM sleep occupies only 22.6% of total sleep time (duration of 2.6 hours), and NREM sleep is the predominant state of sleep with 56.7% of total sleep time (duration of 6.4 hours). As in humans, species that are born immature, such as kittens, rats and rabbits, have the REM sleep state at birth accounting for the majority of sleep duration, with percentages of 100%, 72% and 75%, respectively [60]. Conversely, in animals that are born at an advanced stage of maturity, the proportion of REM sleep is lower than in the aforementioned species. A typical example is the rhesus monkey newborn with REM sleep amounting only 31% of sleep duration [60]. Lambs are born with a certain degree of maturity, with REM sleep in this study representing only 22.6% of total sleep time. The emergence of rumination in our lambs has been shown to influence the architecture of the sleep-wake cycle. As observed in adult ruminants, rumination is predominantly concentrated during the nocturnal phase, being associated with the resting position [13,14]. Results of the present study show that the proportions of rumination during the night and daytime are 28.6 ± 4.5%, and 13.3 ± 2.4%, respectively. In 3-month-old calves, rumination accounted for 40% of total activity [42], and a similar proportion (40%) has also been reported in adult sheep [21].

In the present study, the TST decreased from 11.4 h at birth to 6.9 h when rumination appeared, while the duration of wakefulness ranged from 12.1 h during the first period to 11.9 h during the second one. As for calves (9–10 weeks), which have a TST of 25.2% [42], the lambs in the present study exhibited a close proportion of TST of 29.4% when rumination was acquired. The reduction of the TST after the onset of rumination is due to a decrease in the proportions of light sleep/drowsiness, deep sleep/NREM and REM sleep and mainly attributed to fall in the episode number but not in the duration. Synchronisation of behavioural data with polysomnographic recordings revealed that sleep (drowsiness, NREM and REM sleep) postures in newborn lambs were predominantly in the decubitus position. The association between REM sleep and the decubitus position with the head lying down in a typical position has been demonstrated in several species, including sheep. For instance, the dromedary spends 100% of its REM sleep in sternal decubitus with the head lying down on the ground [46].

In conclusion, the present results demonstrate that newborn lambs show a bimodal pattern of rest-activity and exhibit a significant amount of sleep, both during diurnal and nocturnal hours. As is the case with precocial species, newborn lambs exhibit a greater prevalence of NREM sleep relative to REM sleep. However, after a period of 4–6 weeks, the rumination function becomes apparent and exerts an influence on the lambs’ sleep architecture and rest-activity pattern. This phenomenon is evidenced by a reduction in the duration of rest and the different sleep stages (somnolence, NREM sleep, and REM sleep), while the duration of wakefulness remains unchanged. In certain instances, the synchronization of rumination and SWS was observed, attributable to the weaker muscle development and less well-established rumination exhibited by the lambs. The results of this study suggest that this physiological process in newborn ruminants may interfere or compete with sleep stages without affecting wakefulness.

## Supporting information

S1 File Raw data for lamb vigilance states and behavioral scores. The file (XLSX) contains three sheets: (1) RawData_PhaseI, containing recording data for lambs at birth; (2) RawData_PhaseII, containing recording data for lambs after the onset of rumination; and (3) Abbreviations, providing legend for Lamb_ID, Vigilance_State, and the 0–6 Behavioural_Score scale.
(XLSX)
